# Meniscal Repair Achieves Comparable Mid‐Term Functional Performance to Partial Meniscectomy Following Anterior Cruciate Ligament Reconstruction in Highly Active Patients

**DOI:** 10.1111/os.70419

**Published:** 2026-10-08

**Authors:** Hao Duan, Haiyang Zhang, Jingyi Sun, Xiaohan Zhang, Tao Li, Jingbin Zhou

**Affiliations:** ^1^ Sports Medicine and Rehabilitation Department Beijing Chaoyang Hospital of Capital Medical University Beijing China; ^2^ Key Laboratory of Sports Trauma and Rehabilitation of the General Administration of Sport of China Beijing China

**Keywords:** anterior cruciate ligament reconstruction, functional performance, meniscal repair, partial meniscectomy

## Abstract

**Objective:**

Concomitant meniscal injuries accompany 40%–60% of anterior cruciate ligament (ACL) ruptures. Surgeons often face a clinical choice between meniscal repair, which prioritizes long‐term joint preservation, and partial meniscectomy, which is traditionally perceived to facilitate earlier functional recovery. Despite the biological advantages of repair, persistent concerns remain that its restrictive postoperative protocols might impede mid‐term functional performance compared to meniscectomy. This study aims to compare quadriceps/hamstring strength, dynamic balance, functional hop performance, and patient‐reported outcomes (PROMs) at 2–5 years post‐ACLR between patients undergoing concomitant meniscal repair versus partial meniscectomy.

**Methods:**

This retrospective cohort study evaluated highly active patients who underwent ACLR between 2019 and 2023. Of 68 patients who underwent ACLR with a concomitant meniscal injury and a pre‐injury Tegner score ≥ 6,16 were excluded: 5 due to revision ACLR or secondary meniscal procedures during the follow‐up period, 8 due to concurrent multi‐ligament injuries or full‐thickness articular cartilage defects requiring surgery, and 3 due to knee joint deformities (varus or valgus malalignment > 5°)The remaining 52 patients were stratified into meniscal repair (*n* = 20) or partial meniscectomy (*n* = 32) groups. Outcomes included isokinetic strength (60°/s, 180°/s; Limb Symmetry Index, LSI), Y‐balance test, single‐leg hop (SLH), and PROMs (IKDC, Lysholm, and ACL‐RSI).

**Results:**

At mid‐term follow‐up (mean 4.1 ± 1.2 years for repair, 4.2 ± 1.2 years for meniscectomy; *p* = 0.59), no significant differences existed in strength (quadriceps/hamstring LSI 60°/s and 180°/s), dynamic balance (Y‐balance), hop tests (SLH, SLCH, and SLTH), or PROMs (all *p* > 0.05).

**Conclusion:**

At mid‐term follow‐up (2–5 years), concomitant meniscal repair yielded objective functional performance and patient‐reported outcomes equivalent to partial meniscectomy in highly active patients undergoing ACLR, despite more restrictive early rehabilitation. These findings indicate that the initial postoperative restrictions associated with repair do not result in persistent functional deficits. Although meniscal preservation confers established long‐term chondroprotective advantages, the present analysis addresses functional parity only; the surgical strategy should weigh both mid‐term function and long‐term joint health on an individual basis.

## Graphical abstract

In highly active patients undergoing ACL reconstruction, concomitant meniscal repair yields mid‐term (2–5 years) functional performance, objective strength, and patient‐reported outcomes comparable to partial meniscectomy. These findings support prioritizing meniscal preservation for reparable tears without concern for persistent functional deficits.

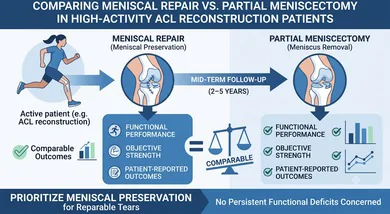

## 1 Introduction

Anterior cruciate ligament reconstruction (ACLR) is the primary surgical intervention for restoring knee stability in active individuals [1, 2]. A significant challenge in ACLR is the high prevalence of concomitant meniscal injuries, which occur in 40% to 60% of cases and necessitate critical intraoperative decisions [3, 4]. Surgeons must often weigh the benefits of meniscal repair, which prioritizes long‐term joint preservation [5], against partial meniscectomy, which is traditionally perceived to offer faster immediate functional recovery [6]. Extensive evidence supports meniscal repair for its superior long‐term outcomes. Successful repair not only mitigates the progression of radiographic osteoarthritis but also significantly reduces long‐term knee pain and preserves patient quality of life at 10‐year follow‐up [7, 8, 9, 10].

However, clinical concerns persist regarding the early functional trade‐offs. The restrictive postoperative protocols associated with repair, such as delayed weight‐bearing, can initially manifest as poorer isokinetic strength and functional jump performance compared to the accelerated protocols of meniscectomy [11, 12, 13].

While short‐term studies (within 1 year) have shown conflicting results regarding recovery speed, there is a distinct lack of objective data focusing on the mid‐term period [11, 14, 15, 16, 17]. The 2–5 years postoperative window is a critical phase where highly active patients aim to consolidate their recovery and sustain pre‐injury activity levels [18, 19]. Although registry‐based data suggests comparable patient‐reported outcomes (PROMs) at 5 years [20], robust evidence comparing objective functional metrics, such as isokinetic strength, dynamic balance, and hop performance, remains scarce during this timeframe. Crucially, emerging biomechanical evidence indicates that partial meniscectomy—particularly medial resection—alters gait mechanics and increases tibiofemoral contact forces, potentially accelerating joint degeneration [21, 22]. Whether these changes manifest as measurable functional deficits in the mid‐term remains unclear.

The purposes of this study were as follows: to compare objective functional outcomes, including isokinetic quadriceps/hamstring strength, dynamic balance (Y‐balance test), and functional hop performance (single‐leg hop, crossover hop, and triple hop), between patients undergoing concomitant meniscal repair versus partial meniscectomy; and to compare functional outcomes, including isokineatient‐reported outcomes (KOOS, Lysholm, IKDC, ACL‐RSI, and postoperative Tegner activity score) between the two groups. We hypothesized that there would be no significant differences in objective functional outcomes or PROMs between the two surgical groups at mid‐term follow‐up.

## 2 Method

### 2.1 Participants

This retrospective cohort study aimed to assess mid‐term functional outcomes in physically active patients following ACLR with concomitant meniscal surgery. We initially screened 68 patients who met the following inclusion criteria: (1) underwent primary ACLR between 2019 and 2023, (2) had a concomitant meniscal injury treated with either repair or partial meniscectomy, and (3) had a pre‐injury Tegner activity score ≥ 6. After applying exclusion criteria, 52 patients were included in the final analysis (Figure 1). Participants were stratified based on the meniscal intervention: meniscal repair (*n* = 20) or partial meniscectomy (*n* = 32). Inclusion required a clinically diagnosed ACL rupture with a concomitant meniscal injury managed by either of the two procedures. To evaluate the baseline functional maintenance of successful index procedures, exclusion criteria included: (1) revision ACLR or secondary meniscal procedures (e.g., secondary meniscectomy due to repair failure) during the follow‐up period; (2) concurrent multi‐ligament injuries or full‐thickness articular cartilage defects requiring surgical intervention; and (3) knee joint deformities, objectively defined as varus or valgus malalignment exceeding 5° measured on full‐leg standing radiographs (hip‐to‐ankle weight‐bearing radiographs). All participants were highly active pre‐injury, defined by a Tegner activity score ≥ 6 [23]. The primary objective was to compare outcomes between groups, including quadriceps and hamstring strength recovery, dynamic balance (Y‐balance test), functional hop performance, and PROMs. Baseline characteristics (age, BMI, and sex) showed no significant intergroup differences (*p* > 0.05). This study was approved by Regional Ethics Committee (202210). Written informed consent was obtained from all participants for the use of their clinical data.

**FIGURE 1:**
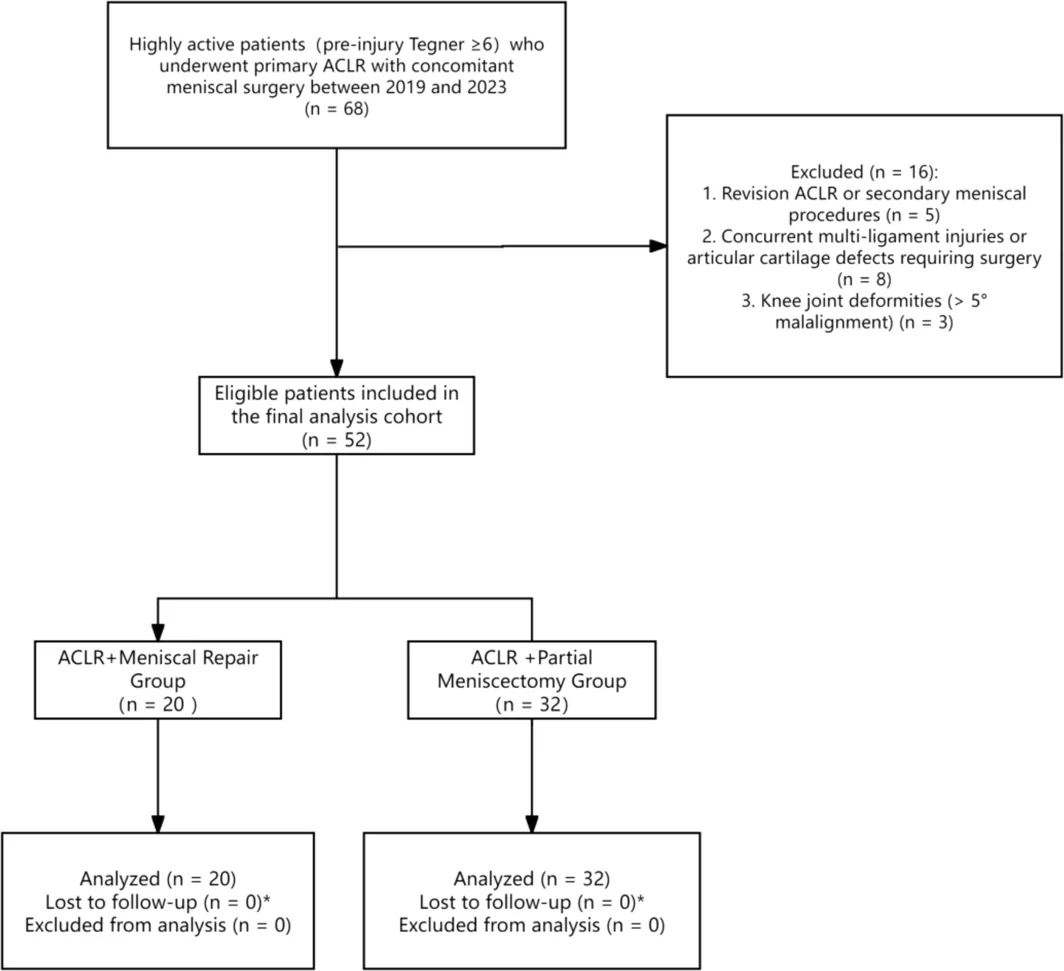
Flow diagram of this study. ACLR, anterior cruciate ligament reconstruction. As this study utilized a cross‐sectional evaluation design at a specific mid‐term follow‐up point, all patients included in the final cohort successfully completed the assessment during their visit. Consequently, there were no cases lost to follow‐up post‐inclusion.

### 2.2 Surgical Management

All participants underwent standardized anterior cruciate ligament reconstruction (ACLR) primarily utilizing autogenous hamstring tendon grafts. Concomitant meniscal injuries were managed intraoperatively based on the specific characteristics of the tear and established surgical principles. For meniscal pathology, repair was performed for reparable tears (e.g., longitudinal‐vertical tears in the vascular zone) using an all‐inside or inside‐out suturing technique. Partial meniscectomy was performed for complex, degenerative, or white‐white zone tears where repair was not feasible. Patients with combined medial and lateral meniscal procedures were categorized based on the more restrictive repair protocol.

Following surgery, all patients adhered to an evidence‐based, phased rehabilitation protocol [24]. Crucially, this protocol incorporated modifications specific to the type of meniscal procedure performed, particularly during the early phases. Phase 1 (Acute: Weeks 0–6) focused primarily on edema control, quadriceps activation, and the restoration of full knee extension. During this stage, significant differences in weight‐bearing and range of motion (ROM) strategies were implemented between the two groups. Patients in the partial meniscectomy group were permitted full weight‐bearing (FWB) as tolerated immediately following surgery without a brace. Progressive ROM was encouraged as pain allowed, with most patients typically achieving full flexion within 2–4 weeks. In contrast, the meniscal repair group followed a more restrictive protocol to protect the suture site. These patients were restricted to non‐weight‐bearing (NWB) or touch‐down weight‐bearing for the first 4 weeks, progressing to partial weight‐bearing (PWB) in weeks 5 and 6. Furthermore, their knee ROM was limited to 0°–90° of flexion for the initial 6 weeks to prevent excessive shear forces on the repair site. Phase 2 (Intermediate: Weeks 6–12) aimed to achieve full knee ROM, enhance quadriceps and hamstring strength, normalize gait patterns, improve dynamic knee control during functional activities, implement general lower body strengthening, and commence aerobic conditioning. By the conclusion of this phase, the protective restrictions imposed on the meniscal repair group (weight‐bearing and ROM limitations) were typically lifted, allowing these patients to progress similarly to the meniscectomy group. Phase 3 (Advanced Strengthening: Months 3–6) emphasized advanced closed and open kinetic chain strengthening exercises, proprioceptive training, and the introduction or progression of foundational sport‐specific skills such as controlled running, landing, jumping, and cutting maneuvers. Phase 4 (Return to Sport/Activity: Months 6+) concentrated on advanced strengthening, power development, agility drills, and intensive sport‐ or activity‐specific skill training. The ultimate goal of this final phase was the safe return to pre‐injury activity levels. Clearance for return to sport was contingent upon meeting predefined functional criteria, including satisfactory limb symmetry indices (LSI > 85%–90%) on hop and strength tests, along with a stable clinical examination.

### 2.3 Isokinetic Strength Testing

Peak knee extensor (quadriceps) and flexor (hamstrings) isokinetic moments were assessed bilaterally using the Biodex System 4 dynamometer (Biodex Medical Systems, USA) at 60°/s and 180°/s. Following a standardized warm‐up and familiarization session supervised by trained testers, subjects were seated with the torso and test leg securely strapped. The knee joint axis was aligned with the dynamometer's axis of rotation. The range of motion was individually set. Testing commenced with the contralateral (healthy) limb followed by the involved limb. Peak torque values were recorded. The Limb Symmetry Index (LSI), calculated as (Peak Torque Involved Limb/Peak Torque Contralateral Limb) × 100%, was used for analysis of both quadriceps (*H*) and hamstrings (*Q*) strength.

### 2.4 Dynamic Balance (Y‐Balance Test)

Dynamic postural control was assessed using the Y‐Balance Test kit (Functional Movement Systems, USA) positioned on a level surface. The kit was configured with a 135° angle between the anterior and each posterior reach direction and a 90° angle between the posterior directions. Participants stood barefoot on the center platform, positioning the test foot with the hallux behind the start line. Hands were placed on the hips. With the stance leg stationary, participants maximally reached with the non‐stance leg in the anterior (A), posteromedial (PM), and posterolateral (PL) directions. The reach distance (cm) was recorded for each direction. After three practice trials per direction, three formal test trials were performed. A trial was invalidated and repeated if balance was lost, the stance foot moved, the reach foot touched down for support, or the reach foot failed to return to the start position. The composite reach score (normalized to leg length) was calculated as: [(*A* + PM + PL)/(3 × Leg length)] × 100%.

### 2.5 Functional Hop Performance

Three hop tests were administered: the single‐leg hop (SLH), single‐leg triple hop (SLTH), and single‐leg crossover hop (SLCH) (Figure 2). After explanation, demonstration, and practice trials for familiarization, participants performed three maximal effort trials for each test on both legs, starting with the contralateral limb. A successful hop required maintaining a stable landing position for at least 2 s. The distance hopped (cm) was recorded. If the third trial exceeded previous distances, a fourth trial was allowed, and the best distance was used. An attempt was discarded if the contralateral limb touched the ground, balance was lost, or an extra hop occurred upon landing. A 30‐s rest was given between trials of the same test, and a 2‐min rest separated different hop tests. Limb symmetry index (LSI) was calculated as (Distance involved limb/Distance contralateral limb) 100% for each hop test.

**FIGURE 2:**
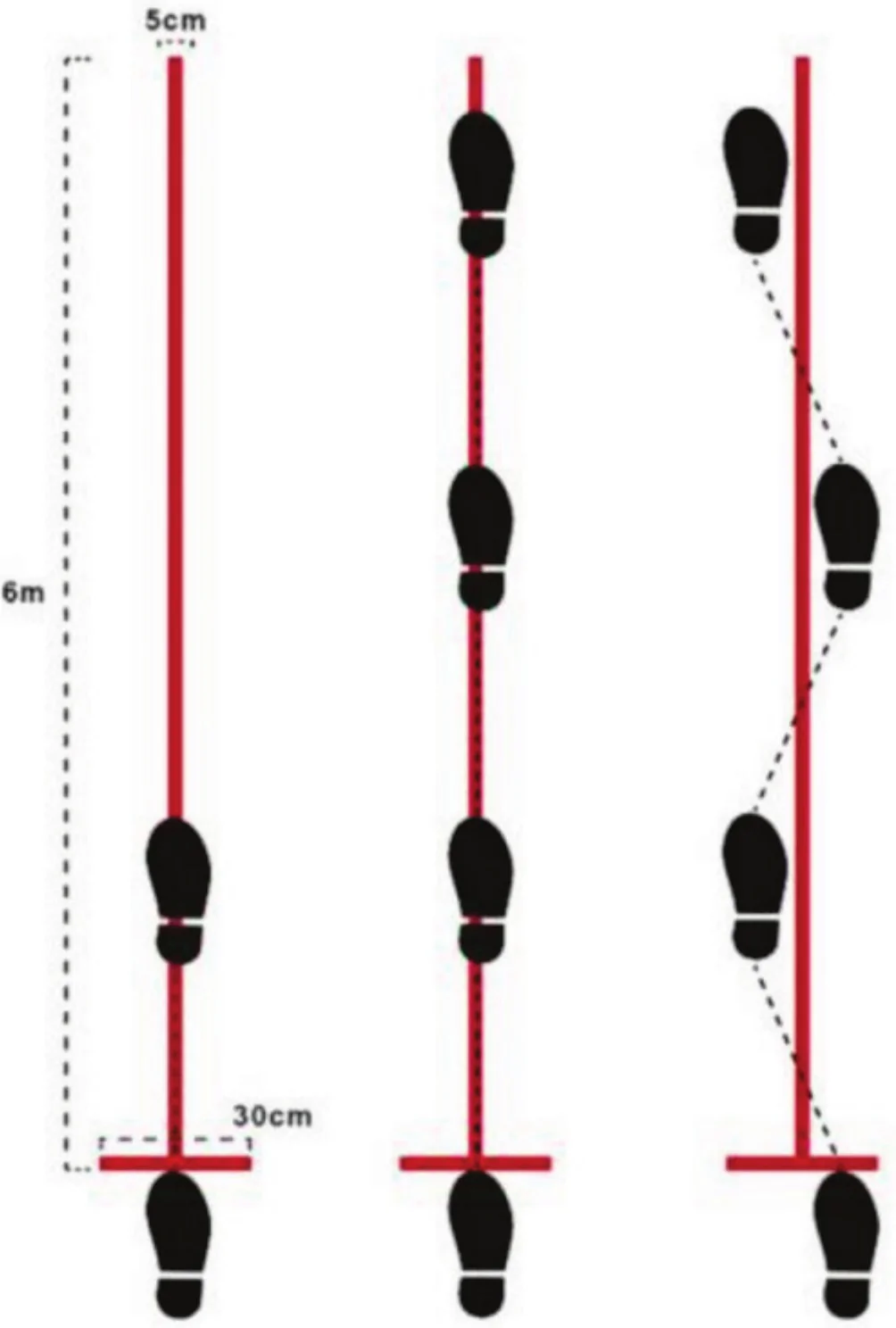
Schematic diagram of hop test [25].

### 2.6 Patient‐Reported Outcomes

To evaluate subjective knee function and patient satisfaction, validated patient‐reported outcome measures (PROMs) were administered at follow‐up. These included the Knee Injury and Osteoarthritis Outcome Score (KOOS) [26], Lysholm Knee Scoring Scale [23], International Knee Documentation Committee (IKDC) subjective knee evaluation [27], ACL‐Return to Sport after Injury (ACL‐RSI) scale [28], and Tegner Activity Scale [23]. Patients completed these questionnaires independently during follow‐up visits under the supervision of a clinical assessor.

### 2.7 Statistical Analysis

Statistical analyses were performed using IBM SPSS Statistics (Version 26.0, IBM Corp., Armonk, NY, USA). Continuous variables were assessed for normality using the Kolmogorov–Smirnov test, supplemented by *P*–*P* and *Q*–*Q* plots. Normally distributed variables are presented as mean ± SD; non‐normally distributed variables as median (interquartile range). Between‐group comparisons of continuous variables were performed with Welch's *t* test (which does not assume equal variances; degrees of freedom were approximated by the Welch–Satterthwaite equation), and categorical variables with Pearson's chi‐square test (or Fisher's exact test when expected cell frequencies were < 5). The corresponding test statistics (*t* or *χ* ^2^) are reported alongside *p* values. A two‐tailed *p* < 0.05 was considered statistically significant.

## 3 Result

### 3.1 Participant Characteristics

The study cohort comprised 52 patients undergoing ACLR with concomitant meniscal surgery, stratified into meniscal repair (*n* = 20) and partial meniscectomy (*n* = 32) groups. Baseline demographics and clinical characteristics demonstrated no significant intergroup differences (Table 1). The repair group averaged 27.3 ± 6.0 years of age versus 29.8 ± 5.8 years in the meniscectomy group (*p* = 0.13). Sex distribution (55% vs. 53% female, *p* = 0.82), BMI (23.8 ± 2.8 vs. 24.7 ± 3.6 kg/m^2^, *p* = 0.35), follow‐up duration (4.1 ± 1.2 vs. 4.2 ± 1.2 years, *p* = 0.59), and pre‐injury Tegner activity scores (6.5 ± 1.3 vs. 6.4 ± 0.7, *p* = 0.64) were statistically comparable, confirming adequate group matching.

**TABLE 1:** Comparison of the general data between the two groups.

	Meniscal repair (*n* = 20)	Partial meniscectomy (*n* = 32)	*p*	Statistic (*t*/*χ* ^2^)
Age (years)	27.3 ± 6.0	29.8 ± 5.8	0.13	−1.60
Female sex, *n* (%)	11 (55)	17 (53)	0.82	0.02 (*χ* ^2^)
BMI (kg/m^2^)	23.8 ± 2.8	24.7 ± 3.6	0.35	−1.10
Follow‐up (years)	4.1 ± 1.2	4.2 ± 1.2	0.59	−0.78
Pre‐injury Tegner	6.5 ± 1.3	6.4 ± 0.7	0.64	0.67

### 3.2 Strength Outcomes

Isokinetic strength testing at 2–5 years postoperatively revealed no statistically significant differences between groups across all evaluated parameters (Table 2). At the lower angular velocity (60°/s), quadriceps limb symmetry index (LSI) averaged 89% ± 11.4% in the repair group versus 80% ± 7.5% in the meniscectomy group (*p* = 0.52). Hamstring LSI at 60°/s was nearly identical between groups (89% ± 5.1% vs. 88% ± 6.0%, *p* = 0.94). At the higher velocity (180°/s), quadriceps LSI values were 87% ± 6.6% (repair) and 90% ± 5.6% (meniscectomy) (*p* = 0.76). Hamstring strength symmetry at 180°/s also showed no intergroup difference, with LSIs of 93% ± 8.1% (repair) and 90% ± 6.1% (meniscectomy) (*p* = 0.73).

**TABLE 2:** A comparison of the strength outcomes between the two groups.

Outcome measure	Meniscal repair (*n* = 20)	Partial meniscectomy (*n* = 32)	*p*	Statistic (*t*/*χ* ^2^)
Strength (60°/s)				
Quadriceps LSI (%)	89 ± 11.4	80 ± 7.5	0.52	0.69
Hamstring LSI (%)	89 ± 5.1	88 ± 6.0	0.94	0.06
Strength (180°/s)				
Quadriceps LSI (%)	87 ± 6.6	90 ± 5.6	0.76	−0.32
Hamstring LSI (%)	93 ± 8.1	90 ± 6.1	0.73	0.36

*Note:* Limb symmetry index (LSI) = (involved limb/contralateral limb) × 100%. Data are expressed as mean ± SD. *p* < 0.05 is considered statistically significant.

### 3.3 Functional Performance

Functional outcomes, including dynamic balance and hop performance, did not differ significantly between groups (Table 3). Y‐Balance composite scores were 100% ± 8.7% (repair) versus 92% ± 1.8% (meniscectomy) (*p* = 0.12). Limb symmetry indices for hop tests were comparable: single‐leg hop (84% ± 4.7% vs. 82% ± 4.0%, *p* = 0.63), crossover hop (90% ± 3.3% vs. 89% ± 2.9%, *p* = 0.78), and triple hop (86% ± 2.8% vs. 88% ± 3.3%, *p* = 0.69).

**TABLE 3:** A Comparison of the functional performance between the two groups.

	Meniscal repair (*n* = 20)	Partial meniscectomy (*n* = 32)	*p*	Statistic (*t*/*χ* ^2^)
Dynamic balance				
Y‐balance composite (%)	100 ± 8.7	92 ± 1.8	0.12	1.70
Hop performance				
Single‐leg hop LSI (%)	84 ± 4.7	82 ± 4.0	0.63	0.58
Crossover hop LSI (%)	90 ± 3.3	89 ± 2.9	0.78	0.41
Triple hop LSI (%)	86 ± 2.8	88 ± 3.3	0.69	−0.60

*Note:* Limb symmetry index (LSI) = (involved limb/contralateral limb) × 100%. Data are expressed as Mean ± SD. *p* < 0.05 is considered statistically significant.

### 3.4 Patient‐Reported Outcomes

PROMs demonstrated no significant differences between groups (Table 4). KOOS scores were 74 ± 17.2 (repair) versus 81 ± 12.4 (meniscectomy) (*p* = 0.10). Lysholm (81 ± 14.4 vs. 82 ± 16.1, *p* = 0.87), IKDC (80 ± 13.4 vs. 81 ± 10.8, *p* = 0.75), and ACL‐RSI (65 ± 20.5 vs. 62 ± 22.1, *p* = 0.60) scores were equivalent. Postoperative activity levels (Tegner: 6.7 ± 0.5 vs. 6.4 ± 0.6, *p* = 0.86) indicated comparable return to pre‐injury function.

**TABLE 4:** Comparison of the results of PROMs of patients in the two groups.

	Meniscal repair (*n* = 20)	Partial meniscectomy (*n* = 32)	*p*	Statistic (*t*/*χ* ^2^)
KOOS	74 ± 17.2	81 ± 12.4	0.10	−1.54
Lysholm	81 ± 14.4	82 ± 16.1	0.87	−0.20
IKDC	80 ± 13.4	81 ± 10.8	0.75	−0.30
ACL‐RSI	65 ± 20.5	62 ± 22.1	0.60	0.53
Post‐operative Tegner	6.7 ± 0.5	6.4 ± 0.6	0.86	1.95

## 4 Discussion

In this cohort of highly active patients with ACL reconstruction and a concomitant meniscal injury, mid‐term follow‐up (2–5 years) revealed no significant differences between meniscal repair and partial meniscectomy in quadriceps or hamstring strength limb symmetry indices, dynamic balance, hop test performance, or any patient‐reported outcome measure (Tables 2, 3, 4). The principal finding is that concomitant meniscal repair does not adversely affect objective functional performance or patient‐reported outcomes at mid‐term. Despite the more restrictive early rehabilitation required to safeguard the healing meniscus, the repair group attained limb symmetry indices in muscle strength, dynamic balance, and hop performance equivalent to the partial meniscectomy group.

### 4.1 Resolution of Early Functional Disparities and Rehabilitation Protocols

Early postoperative disparities in strength and hop symmetry after meniscal repair have been reported, with some investigators observing deficits within the first postoperative year [11, 29]. Contemporary evidence indicates that these disparities largely resolve by 6–12 months [14, 15], and the present mid‐term data demonstrate that any such deficits are fully resolved by 2–5 years. This normalization occurs despite divergent early rehabilitation pathways, underscoring the value of structured, criterion‐based rehabilitation [24]. A central distinction between the two strategies lies in their loading regimens. Partial meniscectomy typically permits early—often immediate—full weight‐bearing, whereas meniscal repair generally mandates a period of protected, frequently non‐weight‐bearing or touch‐to‐toe weight‐bearing with restricted range of motion to protect the healing tissue. Evidence‐based, progressive protocols such as the OPTIKNEE consensus [24] advocate advancing weight‐bearing and strengthening according to objective milestones rather than fixed timeframes. Our finding that repair patients recover functional parity despite this more constrained initial loading argues against unduly prolonged non‐weight‐bearing regimens and supports criterion‐driven progression.

### 4.2 Objective Assessment Beyond Patient‐Reported Outcomes

Historically, much of the literature comparing meniscal repair and meniscectomy has relied on patient‐reported outcome measures such as the IKDC or Lysholm scores [22, 30]. Although PROMs capture perceived function, they often fail to detect subtle biomechanical deficits or side‐to‐side asymmetries evident only through rigorous physical testing [31]. By incorporating isokinetic strength at 60°/s and 180°/s, dynamic balance (Y‐balance test), and a battery of hop tests, the present study provides a more granular, objective assessment. The observed parity in limb symmetry across all metrics suggests that the restrictive early protocols associated with repair do not manifest as persistent neuromuscular or strength deficits at mid‐term.

### 4.3 Long‐Term Chondroprotection and Joint Homeostasis

The clinical value of meniscal repair should also be weighed through the lens of long‐term joint homeostasis. Partial meniscectomy, particularly of the medial meniscus, alters tibiofemoral loading and increases peak contact forces by approximately 15%–25% [19, 21, 32]; such alterations manifest clinically as accelerated cartilage degeneration, and meta‐analytic data indicate meniscectomy is associated with a higher incidence of osteoarthritis than repair [9, 10]. Successful repair preserves the knee's load‐distribution function. The present study confirms that this biological advantage is achieved without sacrificing mid‐term functional performance, reinforcing the paradigm of meniscal preservation whenever surgically feasible [8, 33]. Recent evidence further reports lower failure rates after repair than after meniscectomy [34], supporting the superior survivorship of preservation.

### 4.4 Implications for Clinical Decision‐Making

For the active patient with a reparable concomitant meniscal tear, these findings support a tissue‐preserving strategy when surgical repair is feasible: functional parity with meniscectomy is attained by mid‐term, whereas the established long‐term chondroprotective and survivorship benefits favor preservation [5, 9, 34]. Nevertheless, the decision must be individualized. Repair requires adherence to a protected weight‐bearing protocol and is contingent on tear pattern, tissue quality, and knee stability; irreparable or complex degenerative tears may still warrant meniscectomy. Importantly, the more restrictive early rehabilitation should not be viewed as a barrier to equivalent mid‐term outcomes, but rather as a time‐limited investment in both tissue healing and lifelong joint health. Shared decision‐making should integrate objective functional goals, activity demands, and long‐term osteoarthritis risk.

Several limitations warrant consideration. First, the retrospective design and relatively small sample size (*n* = 52) may lack power to detect subtle subgroup differences. Second, we did not categorize specific meniscal tear patterns, such as ramp or complex bucket‐handle tears, which dictate differing surgical complexity and rehabilitation constraints. Additionally, the absence of early (6–9 months) functional data precludes commentary on the speed of recovery, focusing instead on maintained mid‐term outcomes. Future studies should employ multicenter longitudinal cohorts and advanced imaging to correlate functional maintenance with structural joint integrity beyond 5 years.

## 5 Conclusion

At 2–5 years postoperatively, concomitant meniscal repair yielded objective functional performance and patient‐reported outcomes equivalent to partial meniscectomy in highly active patients undergoing ACLR. These findings indicate that the more restrictive early rehabilitation associated with repair does not result in persistent functional trade‐offs at mid‐term. While the established long‐term chondroprotective and survivorship advantages of meniscal preservation support repair of reparable tears whenever surgically feasible, the present data pertain to mid‐term function and should be interpreted alongside tear‐specific factors and individual patient goals.

## Author Contributions

**Hao Duan:** conceptualization, writing – original draft, methodology, investigation, data curation, software. **Tao Li:** supervision, project administration, writing – review and editing, data curation. **Jingyi Sun:** project administration, resources, supervision, data curation, visualization. **Haiyang Zhang:** conceptualization, methodology, investigation, validation, formal analysis. **Xiaohan Zhang:** conceptualization, project administration, resources, visualization, funding acquisition. **Jingbin Zhou:** writing – review and editing, supervision.

## Funding

The authors have nothing to report.

## Conflicts of Interest

The authors declare no conflicts of interest.

## Data Availability

The data that support the findings of this study are available on request from the corresponding author. The data are not publicly available due to privacy or ethical restrictions.
